# Geraniol as a novel antivirulence agent against bacillary dysentery-causing *Shigella sonnei*

**DOI:** 10.1080/21505594.2017.1412031

**Published:** 2017-12-19

**Authors:** Zainulabedeen R. M. H. Mirza, Thaer Hasan, Veronique Seidel, Jun Yu

**Affiliations:** Strathclyde Institute of Pharmacy and Biomedical Sciences, University of Strathclyde, Glasgow, Scotland, UK

**Keywords:** antivirulence, antibiotic resistance, bacillary dysentery, geraniol, *Shigella sonnei*

## Abstract

Antimicrobial resistance has emerged as a major challenge to modern medicine and it has become urgent to seek alternative approaches to treat infections caused by fast-evolving multi-resistant clones of bacillary dysentery-causing *Shigella sonnei*. Here, we show that geraniol, a natural substance present in the essential oils of plants such as rose and lemongrass, can reduce *S. sonnei* proliferation inside host cells and protect *Galleria mellonella* larvae from killing by *S. sonnei* infection. We present evidence that geraniol competitively inhibits the catalytic activity of the master virulence regulator, DsbA, a periplasmic disulphide bond oxidoreductase required for *Shigella* survival in the host cell cytosol. Our observations suggest that geraniol holds a great therapeutic potential that should be further exploited.

Bacillary dysentery remains one of the most important diarrhoeal diseases affecting humans in the 21st century particularly in resource-poor nations [1]. The etiological agents of this disease, *Shigella* bacteria, derive from diverse origins of *Escherichia coli* [2,3]. *Shigella sonnei* is the causative agent for the current bacillary dysentery pandemic in many of the newly industrialised countries across all continents and has replaced *Shigella flexneri*, once the most prevalent species globally [1]. Contemporary epidemic *S. sonnei* strains mostly belong to the lineage III and exhibit multiple drug resistance due to the spread of transposons and independent point mutations in the gyrase gene [4]. In addition to that, developing an effective vaccine against Shigella remains a challenge [5]. Hence, it has become urgent to seek alternative approaches to treat *Shigella* infection. Here, we show that geraniol, a natural substance present in the essential oils of plants such as rose and lemongrass [6] can reduce *S. sonnei* proliferation inside host cells and protect *Galleria mellonella* larvae from killing by *S. sonnei* infection. We present evidence that geraniol competitively inhibits the catalytic activity of the master virulence regulator, DsbA, a periplasmic disulphide bond oxidoreductase required for *Shigella* survival in the host cell cytosol [7].

It has been previously reported that a natural flavonoid called propolin D could moderately reduce the production of inflammatory cytokines IL-1 and IL-18 from infected macrophage-like U937 cells and inhibit the intracellular proliferation of *S. sonnei* in this cell line as well as in epithelial HEp-2 cells [8]. Following on from this study, here, we treated HEK 293 cells with propolin D for one hour followed by staining with acridine orange. The latter is a dye which emits a red fluorescence upon non-covalent binding to negatively-charged and aromatic molecules [9] such as a flavonoid like propolin D would be once inside HEK 293 cells (pH 7.3) owing to its 3',4'-disubstituted pattern [10]. The heavily stained HEK 293 cells were evidence of a strong accumulation of propolin D intracellularly. In contrast, eriodictyol, a compound that shares identical flavonoid rings but lacks the prenylated (terpene-derived) side chain, showed very little intracellular accumulation (Fig. 1A). Not surprisingly, only supplementation of 42 µM propolin D, but not of 42 µM eriodictyol, in the culture medium was able to inhibit *S. sonnei* growth inside HEK 293 cells (data not shown). These observations strongly suggested that the terpenic side chain was responsible for the accumulation of propolin D, and its activity on *Shigella*, inside host cells. As many terpenes are known to possess antimicrobial activity,[11] this led us to hypothesise that the terpenic side chain could be the key moiety responsible for the inhibition of *S. sonnei* intracellular growth.

**Figure 1.**
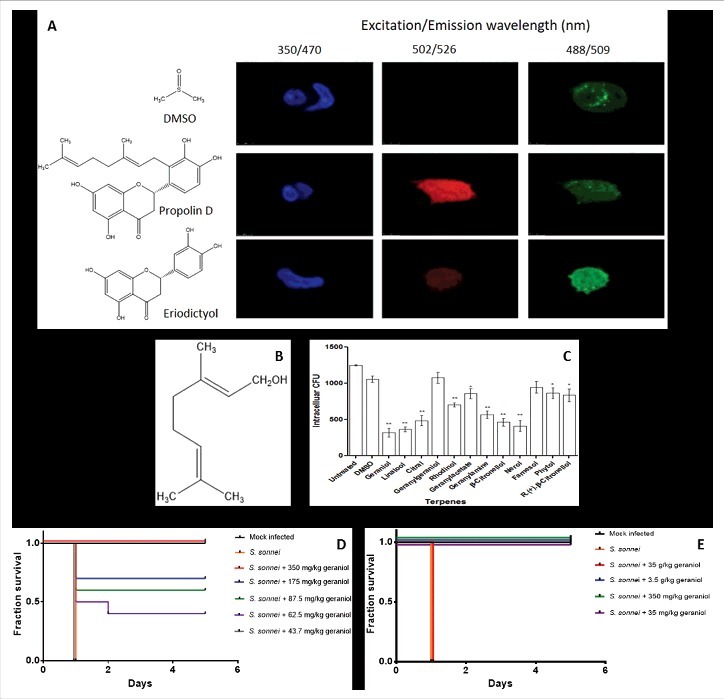
Cellular location of propolin D and functional analysis of geraniol. (A) Propolin D, but not eriodictyol, accumulates inside HEK 293 cells. Cells were treated with propolin D (42 µM), eriodictyol (42 µM) or DMSO (solvent used to solubilise the compounds) for 1 h. Following washes with PBS (3x for 5 min), cells were fixed with 3.7% paraformaldehyde and stained with 10 mM acridine orange for 15 min. Cells were further stained with DAPI (for nuclei) and Alexa Fluor 488® phalloidin (for actin) according to the manufacturer's instructions (Sigma Aldrich, UK). Images were taken using a confocal microscope (Leica Microsystems). Propolin D was detected under excitation at 502 nm and emission at 526 nm; cell nuclei were detected under excitation at 350 nm and emission at 470 nm and actin was detected under excitation at 488 nm and emission at 509 nm. (B) Chemical structure of geraniol. (C) Geraniol significantly reduces *S. sonnei* proliferation inside HeLa cells. The effect of geraniol and other natural terpenes on *S. sonnei intracellular* growth was determined by a gentamicin-killing assay [8]. Cells were infected with *S. sonnei* for 2 h in the presence of terpenes at 42 μM concentrations. The cells were lysed 2 h post-infection with 0.1 Triton X-100 and lysates were plated out on Luria-Bertani agar plates for enumeration of colony forming units (CFU). Experiments were carried out in triplicates and repeated twice. Data shown are pooled means + SD. Differences between untreated and treated groups were assessed using the Student's t-test (* P < 0.05; ** P < 0.01). (D) Geraniol protects *Galleria mellonella* (wax moth) larvae from killing by *S. sonnei*. Experiments were carried out as described using 2–3 days old larvae; 10 larvae per group [12 ]. For infection, 10 µL of a bacterial suspension containing 10^5^ of live *S. sonnei* was injected to the right front leg of each larva. Mock infection was done by injection of PBS. For geraniol protection, geraniol (10 µL) was injected at the indicated doses to the left front leg of each larva. All larvae were observed for 5 days. Larvae death is indicative by melanisation and loss of mobility. Experiments were repeated three times (*n*=3) and pooled data were used to generate the graphs. Differences between *Shigella*-infected untreated and treated groups were assessed using the Gehan-Breslow-Wilcoxon test with a significance value set at P < 0.05. (E) Cytotoxicity of geraniol to *Galleria mellonella* larvae. Each group contained 10 larvae as in D, geraniol (10 µL) was injected at the indicated doses to the front leg of each larva. A mock treatment was done using PBS. All observations were done as in D. Differences between treated groups and the mock-infected control were assessed using the Gehan-Breslow-Wilcoxon test with a significance value set at P < 0.05.

To test this hypothesis, we examined twelve terpenes for their potential in inhibiting intracellular *S. sonnei* growth using a gentamicin-killing assay [8]. The monoterpene geraniol (Fig. 1B) was found to be the most potent among the tested compounds, with as little as 42 µM significantly inhibiting intracellular bacterial growth (P < 0.01) (Fig. 1C). We further exploited a *Galleria mellonella* larvae model developed as a simple *in vivo* model to assess *Shigella* virulence [12]. As shown in Fig. 1D, the injection of 10^5^ of *S. sonnei* bacteria killed all 10 larvae in one day while the use of geraniol at doses of 350, 175, 87.5 and 62.5 mg/kg significantly improved larval survival compared to *Shigella*-infected untreated larvae (P < 0.05). We observed that the use of 62.5 mg/kg of geraniol could significantly protect 40 % of larvae for 5 days (P = 0.0118) and that all larvae were completely protected for 5 days when treated with 350 mg/kg of geraniol (P < 0.0001). We also used the *Galleria mellonella* larvae model to examine the cytotoxicity of geraniol and found that all larvae survived for 5 days when up to 3.5 g/kg of geraniol was administered per larvae (Fig. 1E). However, compared to the mock-infected control, all larvae were killed in one day when 35 g/kg of geraniol was given per larvae (P < 0.0001), which had the same killing capacity as a *S. sonnei* infection at 10^5^ bacteria per larvae. Thus, the larvae tolerance level was at least 10-fold higher than the therapeutic level required to control *S. sonnei* infection.

Propolin D has previously been shown to possess potent activity against *Staphylococcus aureus* but no direct activity against a Gram-negative bacterium such as *Pseudomonas aeruginosa* [13]. We found that this was also true for geraniol, which had little direct inhibition on *S. sonnei* growth when tested using a microdilution assay [14] in a rich broth such as Luria*-*Bertani and in a minimal M9 broth (MIC > 500 mg/L). These facts strongly suggested that propolin D, as well as geraniol, could either enhance a host defence mechanism responsible for controlling intracellular bacteria or target a bacterial factor only active inside the host cell cytosol where *Shigella* thrives [15].

Previous studies revealed that the *Shigella* periplasmic disulphide bond oxidoreductase, DsbA, is vital in supporting the survival of *S. flexneri* in the host cell cytosol [7]. The latter is a highly reducing environment with high concentrations of reduced glutathione (GSH) because the cytosolic thioredoxin-1 and reduced glutathione (GSH)/oxidised glutathione (GSSG) redox couples are not in equilibrium [16]. We hypothesised, here, that *Shigella* uses DsbA to convert excessive GSH to GSSG, as a way to overcome the stress caused by the highly reducing cytosolic environment and thus survive and grow inside cells [7]. We investigated if geraniol could inhibit the enzymatic activity of *Shigella* DsbA *in vitro* and inside host cells.

Although DsbA is fundamentally a disulphide bond catalyst,[17] it does exhibit reduction activity in a scrambled ribonuclease assay similarly to the mammalian protein disulphide isomerase (PDI) [18]. We first performed an *in vitro* assay using fluorescent di-eosin-glutathione disulfide (Di-E-GSSG) according to a previously published methodology [19]. Di-E-GSSG is not brightly fluorescent in its disulfide form due to self-quenching, but reduced eosin-glutathione (E-GSH) formed upon reduction of the disulphide bond by PDI, emits strong fluorescence at 545 nm when excited at 525 nm [19]. In order to use DsbA in this assay, we purified the wild type *Shigella* DsbA protein as well as a mutant DsbA33G protein that had a substitution of cysteine 33 for glycine 33 at the active site [20]. As shown in Fig. 2A, the wild type DsbA protein was able to effectively convert Di-E-GSSG to a more fluorescent E-GSH with an estimated Vmax of 14.97 ± 5.39 RFU/s whereas the mutant protein DsbA33G had a Vmax of 10.14 ± 3.44 RFU/s. Importantly, in the presence of 42 µM of geraniol, the wild type DsbA and DsbA33G both had significantly reduced Vmax values (13.89 ± 5.05 RFU/s and 9.03 ± 3.44 RFU/s, respectively). These data demonstrated that glutathione is indeed a substrate for *Shigella* DsbA, and that geraniol may inhibit the reductive activity of DsbA on Di-E-GSSG. To further characterise the type of inhibition involved, we produced Lineweaver-Burk plots in the presence and absence of geraniol for DsbA and DsbA33G (Fig. 2B and Fig. 2C, respectively). It was clear that geraniol competitively inhibited the DsbA-catalysed reduction of Di-E-GSSG as the Y-intercept (1/V) was not changed but the line was shifted to the right on the X-axis (1/S) in the presence of geraniol. The Km values for DsbA were estimated as 200.3 and 331.8 nM, in the absence and presence of 42 µM geraniol, respectively (Fig. 2B). Interestingly, Km values for DsbA33G were estimated as 537.6 and 331.8 nM in the absence and presence and of 42 µM geraniol, respectively (Fig. 2C). This is consistent with the fact that, at low concentrations of Di-E-GSSG, an addition of geraniol moved the curve to the left on the X-axis (1/S) and accelerated the reaction of DsbA33G (arrow in Fig. 2A).

**Figure 2.**
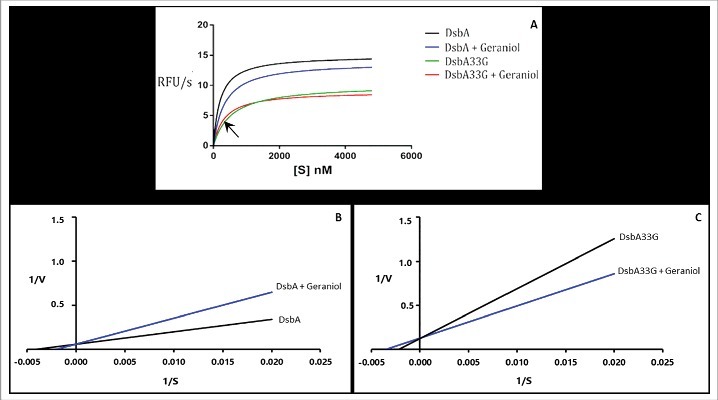
Reduction of Di-E-GSSG by DsbA proteins *in vitro*. (A) Reducing activity of wild type DsbA and DsbA33G in the presence and absence of geraniol. DsbA or DsbA33G (40 nM) was incubated with various concentrations of Di-E-GSSG (50 nM - 5 µM) in a DsbA assay buffer at room temperature according to a previously published method [19]. Fluorescence was measured with excitation at 525 nm and emission at 545 nm using a Spectramax microplate reader M5 (Molecular device). Conversion of Di-E-GSSG was expressed as relative fluorescence unit per second (RFU/s). Theoretical hyperbolic curves allowed the estimation of the Vmax for wild type DsbA and for DsbA33G (14.97 ± 5.39 and 10.14 ± 3.44 RFU/s, respectively). In the presence of 42 µM of geraniol, the Vmax values were significantly reduced to 13.89 ± 5.05 and 9.03 ± 3.44 RFU/s, respectively (P = 0.0052 and 0.0069, respectively). Error bars were removed for clarity. Km values were estimated using the Lineweaver-Burk plot for DsbA (B) and DsbA33G (C). Km was estimated as 200.3 and 331.8 nM for DsbA without and with 42 µM geraniol, respectively. Km was estimated as 537.6 and 331.8 nM for DsbA33G without and with geraniol, respectively. All experiments were repeated three times (*n *= 3) in triplicates each time. Data shown are pooled means + SD. Statistical significance was calculated using the Bonferroni correction t test with a significance value set at P < 0.01.

The above data have demonstrated that glutathione is a substrate for *Shigella* DsbA. However, a fundamental question remains unanswered: does *Shigella* DsbA catalyse reduced GSH to oxidised GSSG, which is hypothesised to be vital for *Shigella* to survive and proliferate in the host cell cytosol [7]. As no *in vitro* assay has been described, we set up an *in vivo* assay using fluorescent E-GSH. Weakly fluorescent Di-E-GSSG was completely reduced with 10 mM DTT to highly fluorescent E-GSH. We then added 75 nM of E-GSH to Luria*-*Bertani broth that was used to culture a wild type as well as a mutant *Shigella* strain in which the *dsbA* gene was deleted (*ΔdsbA*). As the wild type strain grew, the fluorescence of the culture gradually decreased, from 5,000 to 2,000 RFU, over time (Fig. 3A). In contrast, the culture of the *ΔDsbA* strain showed no reduction in fluorescence although the mutant strain grew well under such mildly reducing conditions (Fig. 3B). As anticipated, the complemented strain *ΔDsbA/*pDsbA showed the same ability in reducing the culture fluorescence, from 5,000 to 2,000 RFU, over time, presumably due to the over expression of DsbA from the plasmid (Fig. 3C). Importantly, geraniol (42 µM) inhibited the wild type as well as the complemented strains in conversion of E-GSH to Di-E-GSSG; the reduction of fluorescence did not go below 4000 RFU (Fig. 3A, 3B). To our knowledge, this is the first time that *Shigella* DsbA has been shown to catalyse GSH to GSSG, which strongly support the hypothesis that catalysis of GSH to GSSG in the host cell cytosol is imperative for *Shigella* to survive, proliferate and establish infection [7].

**Figure 3.**
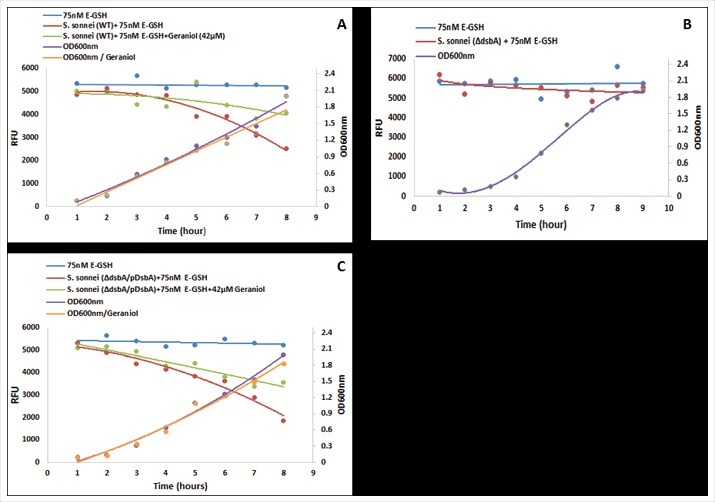
Analysis of DsbA enzymatic activity *in vivo*. Wild type (A), *ΔdsbA* (B) and complemented (*ΔdsbA* /pDsbA) (C) *Shigella sonnei* strains were grown in LB-broth supplemented with E-GSH (75 nM) in the absence and presence of geraniol (42 µM). Bacterial growth was measured by optical density (OD600 nm; right axes) over time. The formation of Di-E-GSSG was measured by relative fluorescence units (RFUs; left axes) with excitation at 525 nm and emission at 545 nm using a Spectramax microplate reader M5 (Molecular device) over time. The experiments were repeated twice (*n *= 2) in triplicates each time and pooled data were used to generate the graphs. Error bars were removed for clarity.

Antimicrobial resistance has emerged as a major challenge to modern medicine and public health and fast-evolving multi-resistant clones are the cause of the current S. sonnei pandemic [4]. The chilling fact is that antimicrobial resistance will occur to any new antibiotic sooner or later. The major advantage of targeting virulence in intracellular pathogens, is that there is a very remote possibility for resistance mechanisms to develop [21].

DsbA is present in all Gram-negative bacteria and some Gram-positive bacteria, including in many pathogens of human and animals. DsbA is considered as a master virulence regulator as it catalyses the formation of disulphide bond formation in exported proteins, of which many are virulence factors [22]. Targeting Dsb proteins for anti-virulence therapy is not new but previous studies focusing on disrupting DsbA-DsbB interaction have yielded little progress; DsbB is required for recycling the non-functional reduced DsbA to its functional oxidised form [22]. Our study presents for the first time a way of controlling *S. sonnei* infection *via* inhibition of DsbA inside host cells. Our observations may also be relevant to control other intracellular pathogens such as *Francisella tularensis* that thrive in the host cytosol and require DsbA [23]. It is also of interest to note that previous studies have showed that geraniol can modulate the host immune system for the production of anti-inflammatory cytokines such as IL-10,[24] which could be of particular interest in bacillary dysentery where there is hyper-inflammation. We have also gathered some additional (unpublished) evidence that geraniol is able to inhibit the growth of adhesive and invasive *Escherichia coli* inside macrophages. Such bacteria have been identified as a key aetiological factor to Crohn's disease, another hyper-inflammatory condition affecting the bowel [25]. Additionally, geraniol has been shown to work in synergy with antibiotics [26]. Taken altogether, these observations indicate that geraniol holds a great therapeutic potential that should be further exploited.

## Abbreviations

*Di-E-GSSG*: Di-eosin-glutathione disulfide
*DMSO*: Dimethylsulfoxide
*E-GSH*: Eosin-glutathione monosulfide
*GSH*: Reduced glutathione
*GSSG*: Oxidised glutathione
*HEK*: Human Embryonic Kidney
*IL*: Interleukin
*PBS*: Phosphate-buffered saline
